# The Vaginal Microbiota Among Adolescent Girls in Tanzania Around the Time of Sexual Debut

**DOI:** 10.3389/fcimb.2020.00305

**Published:** 2020-06-25

**Authors:** Suzanna Carter Francis, Tania Crucitti, Tom Smekens, Christian Holm Hansen, Aura Andreasen, Vicky Jespers, Liselotte Hardy, Julia Irani, John Changalucha, Kathy Baisley, Richard Hayes, Deborah Watson-Jones, Anne Buvé

**Affiliations:** ^1^MRC Tropical Epidemiology Group, London School of Hygiene and Tropical Medicine, London, United Kingdom; ^2^Department of Clinical Sciences, Institute of Tropical Medicine, Antwerp, Belgium; ^3^Department of Public Health, Institute of Tropical Medicine, Antwerp, Belgium; ^4^Mwanza Intervention Trials Unit, National Institute for Medical Research, Mwanza, Tanzania; ^5^MRC/UVRI and LSHTM Uganda Research Unit, Entebbe, Uganda; ^6^Department of Clinical Research, London School of Hygiene and Tropical Medicine, London, United Kingdom; ^7^Belgian Health Care Knowledge Centre, Brussels, Belgium; ^8^National Institute for Medical Research, Mwanza, Tanzania

**Keywords:** bacterial vaginosis, vagina microbiota, Africa, biofilm, *Lactobacillus crispatus*, *Gardnerella vaginalis*, sialidase, adolescence

## Abstract

The aetiology of bacterial vaginosis (BV) is not well-understood, and prevalence appears to be higher among women living in sub-Saharan Africa. A recent conceptual model implicates three main bacteria (*Gardnerella vaginalis; Atopobium vaginae;* and *Prevotella bivia*), sexual activity, sialidase activity, and biofilm formation in the pathogenesis of BV. We describe the vaginal microbiota, presence of the putative sialidase A gene of *G. vaginalis*, and biofilm among 386 adolescent girls aged 17 and 18 years in a cross-sectional study in Mwanza, Tanzania around the time of expected sexual debut. Vaginal swabs were collected and tested by quantitative polymerase chain reaction (qPCR) for five *Lactobacillus* species, *G. vaginalis, A. vaginae, P. bivia*, the sialidase A gene of *G. vaginalis*, and by fluorescence *in situ* hybridisation (FISH) for evidence of *G. vaginalis* and *A. vaginae* biofilm. We conducted a risk factor analysis of *G. vaginalis, A. vaginae* and *P. bivia*, and explored the associations between biofilm, the presence of the sialidase A gene, and non-optimal vaginal microbiota (Nugent 4–7)*. L. crispatus* and *L. iners* were detected in 69 and 82% of girls, respectively. The prevalence of *L. crispatus* was higher than previously reported in earlier studies among East and Southern African women. *G. vaginalis, A. vaginae, P. bivia* were independently associated with reported penile-vaginal sex. Samples with all three BV-associated bacteria made up the highest proportion of samples with Nugent-BV compared to samples with each bacterium alone or together in pairs. Of the 238 girls with *G. vaginalis*, 63% had the sialidase A gene detected, though there was no difference by reported sexual activity (*p* = 0.197). Of the 191 girls with results for sialidase A gene and FISH, there was strong evidence for an increased presence of sialidase A gene among those with evidence of a biofilm (*p* < 0.001). There was a strong association between biofilm and non-optimal microbiota (aOR67.00; 95% CI 26.72–190.53). These results support several of the steps outlined in the conceptual model, although the role of sexual activity is less clear. We recommend longitudinal studies to better understand changes in vaginal microbiota and biofilm formation around the time of sexual debut.

## Introduction

Vaginal microbiota plays an important protective role in the female reproductive tract. Optimal vaginal microbiota is dominated by lactic acid producing bacteria (*Lactobacillus spp*) which maintains a low pH in the vaginal niche. Bacterial vaginosis (BV), an example of non-optimal vaginal microbiota, is characterised by the loss of protective *Lactobacillus spp*. (*L. crispatus*, as well as *L. gasseri, L. jensenii*, and *L. vaginalis)* and a high relative abundance or load of facultative and/or obligate anaerobes, resulting in the breakdown of the protective mucin layer and inflammation (McKinnon et al., 2019). BV is associated with adverse urogenital and reproductive health outcomes including an increased risk of HIV acquisition (Low et al., 2011; Buvé et al., 2014; Eastment and Mcclelland, 2018). While BV and BV-associated bacteria have been well-described, it is not well-understood how the high abundance of BV-associated bacteria is established and maintained, and how BV develops and resolves (van de Wijgert et al., 2014). Sexual activity has been strongly associated with BV; however, it is not clear whether it is a sexually transmitted or sexually enhanced condition (Fethers et al., 2008; Verstraelen et al., 2010). Describing the vaginal microbiota around the time of sexual debut may offer important insights for understanding the pathogenesis of BV.

*Lactobacillus spp*.-dominated vaginal microbiota is associated with health; however, not all *Lactobacilllus spp*. are equally protective. *L. crispatus*, as well as *L. gasseri, L. jensenii*, and *L. vaginalis*, have been associated with health; while *L. iners* is often found among women with BV (van de Wijgert et al., 2014). A recent prospective study among South African adolescent girls showed that *L. crispatus*-dominated vaginal microbiota, not *L. iners*, was associated with a decreased risk of acquiring HIV (Gosmann et al., 2017). In addition, this study reported that only 10% of women had microbiota dominated by *L. crispatus*, while 32% were dominated by *L. iners*. This is in contrast to a study conducted among Caucasian women in high income countries 45% of whom had *L. crispatus*-dominated vaginal communities (Ravel et al., 2011). Several studies have reported that Black African and African-American women compared to Caucasian or Asian women are less likely to carry *L. crispatus*, and more likely to carry *L. iners* (van de Wijgert et al., 2014). It has been hypothesised that such differences in the vaginal microbiota may partly explain differences in prevalence of BV between different populations with the highest prevalences found in women in sub-Saharan Africa (Kenyon et al., 2013; Buvé et al., 2014).

In 2019, Muzny et al. presented a conceptual model that implicated three main bacteria and their interactions in the pathogenesis of BV: *Gardnerella vaginalis; Atopobium vaginae;* and *Prevotella bivia*. The model postulated that that virulent strains of *G. vaginalis* are acquired by sexual transmission which adhere to the host epithelium, displace lactobacilli and create a biofilm (Muzny et al., 2019), a structured community of bacteria in a self-produced extracellular matrix which sequesters bacteria making it difficult to treat (Hardy et al., 2017a). Recently it has been shown that different strains of *G. vaginalis* may explain differences in virulence (Vaneechoutte et al., 2019). For example, some, but not all, strains can produce sialidase, which facilitate the destruction of the protective mucin layer on the vaginal epithelium (Lopes Dos Santos Santiago et al., 2011). After this first step, the Muzny model proposes that the synergistic effect of *P. bivia* and *G. vaginalis* enhances growth of both bacteria which both produce sialidase and loss of the mucin layer of the vaginal epithelium. Next, the loss of the mucin layer leads to increased adherence of other BV-associated bacteria, including *A. vaginae*. *A. vaginae* has been shown to elicit a stronger immune response than *G. vaginalis*, and may also have higher resistance to BV treatments such as metronidazole.

We recently published a paper showing a BV prevalence of 25% among girls attending secondary school in Tanzania. In this study the BV prevalence among girls who reported no penile-vaginal sex was 19% compared to 33% in girls reporting having had penile-vaginal sex (Francis et al., 2018). These data strongly suggest that penile-vaginal sex increases the risk for BV. In this paper, we describe the vaginal microbiota from the same study, including the results of quantitative PCR tests for *L. crispatus, L. gasseri, L. jensenii, L. iners, L. vaginalis, G. vaginalis, A. vaginae* and *P. bivia*; putative sialidase A gene of *G. vaginalis* in specimens containing *G. vaginalis*; and BV-associated biofilm dominated by *G. vaginalis* and *A. vaginae* by fluorescence *in situ* hybridisation (FISH). We investigate factors associated with the presence of *G. vaginalis, A. vaginae* and *P. bivia*, and the association between biofilm, putative sialidase gene of *G. vaginalis* and non-optimal microbiota.

## Materials and Methods

The enrolment of the study population and the study procedures have been described in detail elsewhere (Francis et al., 2018). In brief, all government secondary schools in Mwanza city, north-western Tanzania, were mapped and 26 schools with more than 25 girls aged 17 and 18 years were identified and asked to collaborate in the study. The parents of all girls aged 17 and 18 years in forms 1–3 were informed about the study and asked for their informed consent for their daughter to participate in the study if she was <18 years old. The girls were asked for their assent/consent. Assenting/consenting girls were invited to a research clinic where they were interviewed and samples of urine, blood and vaginal fluid were taken for testing for sexually transmitted and reproductive tract infections (STIs/RTIs) and characterisation of the vaginal microbiota. Non-pregnant girls were taught how to self-collect vaginal swabs. They were asked to collect five sequential swabs in the presence of a nurse who provided assistance if needed.

### Laboratory Tests

Laboratory testing was performed according to standard operating procedures. Urine samples were tested for pregnancy using the QuickVue+ Test (QUIDEL, USA). Serum samples were used to test for IgG antibodies for HSV-2 by ELISA (Kalon Biological Ltd., UK). Syphilis was determined by the Immutrep Rapid Plasma Reagin test (Omega Diagnostics, Scotland) and the Treponema pallidum particle agglutination assay (SERODIA, Fujirebio Inc., Japan).

All blood samples were screened with the Determine HIV 1/2 rapid test (Alere, Japan). Reactive samples were tested with the Uni-GoldTM HIV rapid test (Trinity Biotech, Ireland). If both tests were reactive, the final result was deemed positive. If the Uni-Gold test was not reactive, the sample was tested with the HIV 1/2 Stat-Pak test (Chembio, USA). The final result was considered positive if the Stat-Pak result was reactive.

Two flocked swabs (Copan, USA) were eluted in 1.2 mL of diluted phosphate buffered saline (dPBS) (pH 7.4–1:9, PBS:saline), pooled and aliquots were prepared. One aliquot was used to test for *Neisseria gonorrhoeae, Chlamydia trachomatis* and *Mycoplasma genitalium* by in-house PCR (Chen et al., 2008; Hopkins et al., 2010). A second aliquot was used to test for human papillomavirus (HPV) using the Roche Linear Array HPV Genotyping Test (Roche, USA), which detects 37 HPV high and low risk genotypes. A third aliquot was used to assess the presence and concentrations of *L. crispatus, L. gasseri, L. jensenii, L. iners, L. vaginalis, G. vaginalis, A. vaginae* using a qPCR as previously described (Jespers et al., 2012), with the exception of DNA extraction where another method, the QIAmp DNA mini kit, was applied. A fourth aliquot was used for the determination of the bacterial load of *P. bivia* and the detection of the putative sialidase A gene of *G. vaginalis* in specimens containing *G. vaginalis*. DNA was extracted using the Abbott m2000sp automated extraction platform (Abbott Laboratories, USA) according to the manufacturer's instructions for the plasma custom program and incorporating an extra lysis step (Crucitti et al., 2018). For the quantification of *P. bivia*, an in-house qPCR assay was performed targeting the mucin-desulfating sulfatase (*mdsC*) gene. The 25 μL PCR mixture consisted of 12.5 μL Sybr Green master mix (Qiagen, Germany), 0.5 μL of 10 μM of each primer (forward primer: PBsulF 5'ACGTTTGGGCAAAGCTCCTTGTCT3', reverse primer: PBsulR 5'GCGTGTACGCCAGTTGCAAGA3), 6.5 μL Rnase free water, and 5 μL of DNA extract (Lopes dos Santos Santiago et al., 2012). The amplification comprised an initial heating of 95°C for 5 min followed by 45 cycles of 95°C for 5 s and 65°C for 10 s. For samples containing *G. vaginalis*, a qPCR for the putative *G. vaginalis* sialidase A gene was performed as previously described (Hardy et al., 2017b). All qPCRs were run in duplicate and the organism concentrations were expressed as genome equivalents per ml (geq/ml) and log_10_-transformed.

A cotton-tipped vaginal swab was used to prepare two slides. The first slide was Gram stained and examined for vaginal yeast and for BV using the Nugent score for diagnosing BV (Nugent et al., 1991). A Nugent score of 0–3 indicated normal microbiota, 4–6 indicated intermediate microbiota, and 7–10 indicated BV. A Superfrost Plus® slide (Menzel-Gläser, Germany) was heat fixated and stored at room temperature until shipment to the Institute of Tropical Medicine in Antwerp, Belgium (ITM) where it was re-fixated with a Carnoy solution (6:3:1, ethanol:chloroform:glacial acetic acid) for at least 12 h. For all participants who had *G. vaginalis* detected by qPCR, the slide was examined using Peptide Nucleic Acid (PNA)-FISH employing species-specific probes for *A. vaginae* (AtoITM1) and *G. vaginalis* (Gard162), and the broad-range BacUni-1 probe. Procedures and definitions of observations were applied as described elsewhere (Hardy et al., 2015). The Superfrost Plus® slide was not available for the first few weeks of the study; therefore, not all participants have results for the FISH examination. The same swab was inoculated in an InPouch™ TV culture device (BioMed Diagnostics, USA), incubated at 37°C and read every other day for the presence of motile trichomonads for 5 days or until positive.

All laboratory tests were carried out at the Mwanza Intervention Trials Unit at the National Institute of Medical Research (NIMR/MITU) laboratory in Mwanza with the exception of the qPCR on *P. bivia*, the putative sialidase A gene tests on *G. vaginalis* and the visualization of *G. vaginalis* and *A. vaginae* by FISH which were carried out at ITM. The primers and probes at NIMR were ordered from Eurogentec S.A. (Belgium) and PCRs were run on the QIAGEN Rotorgene Q. The primers and probes used at ITM were synthesized by Integrated DNA Technologies (IDT), Illinois, USA, and amplifications were performed using the Corbett Life Science Rotor-Gene^TM^ 6000 (Qiagen, The Netherlands). In order to assess the variability in the results of the qPCR a random selection of 100 stored samples were re-tested at ITM. Initial qPCR results for *L. crispatus* carried out at NIMR/MITU were higher than expected; therefore, we repeated *L. crispatus* qPCR at ITM and compared results. There was less than a log difference between the geometric means between the NIMR/MITU and ITM results (8.72 vs. 7.76, respectively); therefore, we used the NIMR/MITU results in the analysis described below.

### Data Management and Statistical Analysis

Pen and paper questionnaire data and laboratory worksheets were double entered into OpenClinica LLC (Akaza Research, USA). The data were analysed using R version 3.6.3.

Two parameters were analysed for each bacterial species: percentage of girls in which the bacterial species was detected and, if the species was present, the concentration of the bacteria expressed as geq per ml. The presence of the different bacterial species in girls who reported no penile-vaginal sex was compared to that in girls reporting having had penile-vaginal sex using chi-squared tests, and for those in whom the species was present, the mean log_10_ concentrations were compared using *t*-tests. In order to allow for the effect of STIs/RTIs on the vaginal microbiota, the same analyses were repeated excluding the girls with STIs/RTIs (vaginal yeast, HSV-2 infection, gonorrhoea, chlamydial infection, *M. genitalium* infection, HPV, trichomoniasis, syphilis, and HIV). Lastly, the presence and concentrations of the different species were compared in girls in the different categories of Nugent score (1–3, 4–6, 7–10), stratified by reported penile-vaginal sex.

A hierarchical approach was used for the risk factor analysis for each of the BV-associated bacteria: *G. vaginalis; A. vaginae;* and *P. bivia*. We first estimated the crude and independent effects of the socio-demographic characteristics on the presence of the bacteria (level 1). Socio-demographic variables included age, socioeconomic status (SES) and if the participant lived with a parent. Socioeconomic status (SES) was estimated using an indicator based on the type of possessions owned by the head of the household. The independent effects were estimated using multivariable logistic regression adjusted for any other socio-demographic variables with a *p*-value of <0.10. This procedure was repeated for the analysis of behavioural risk factors (level 2), except that the multivariable model at this level was adjusted for not only other behavioural factors with adjusted associations with *p* < 0.10, but also the socio-demographic characteristics that were found to be independently associated with BV from the first stage of the analysis. Behavioural factors that were explored in all girls included menstrual hygiene management, intravaginal cleansing, direction of cleaning after defecation, sexual touching with hands, receptive oral sex and life-time number of sexual partners for penile-vaginal sex. In girls who reported that they had had penile-vaginal sex the same behavioural variables were explored as well as condom use with last partner and age of first sexual partner. *P*-values were obtained with likelihood ratio tests.

Samples tested by FISH were categorised into five groups based on the presence and absence of dispersed and/or adherent *G. vaginalis* and *A. vaginae*: no *G. vaginalis* or *A. vaginae* visualised; dispersed *G. vaginalis* only, no *A. vaginae*; dispersed *G. vaginalis* and *A. vaginae;* adherent *G. vaginalis* only, no *A. vaginae*; and a combination of *G. vaginalis* and *A. vaginae* dispersed and adherent. The latter category included all samples with both *G. vaginalis* and *A. vaginae*, and with at least one bacterium showing adherence. We considered the last two categories with adherent bacteria to be indicative of biofilm. We compared these categories by Nugent score and reported penile-vaginal sex using the Chi squared test for trend.

Samples in which *G.vaginalis* was found were characterised by the presence of the sialidase A gene using two parameters: percentage of girls in which the sialidase A gene was present and, if the sialidase A gene was present, the concentration of the sialidase A gene expressed as geq per ml. The presence of the sialidase A gene and the mean log_10_-transformed concentrations were compared by Nugent score, reported penile-vaginal sex and results of the FISH, again using chi-squared tests and *t*-tests, respectively.

The associations between the presence of the sialidase A gene and biofilm, and between biofilm and non-optimal vaginal microbiota were explored. We combined Nugent score 4–10 to make vaginal microbiota status binary and defined the categories 0–3 as optimal microbiota and 4–10 as non-optimal microbiota. Likewise, we combined the FISH categories to make a binary variable: the first three categories as described above were defined as non-adherent bacteria, and the last two categories were defined as adherent bacteria indicative of biofilm. Crude odds ratios were calculated for the association between detection of the sialidase A gene and microbiota, and then adjusted for the FISH results. Likewise, the crude OR was calculated for the association between FISH results and microbiota, and then adjusted for detection of the sialidase A gene.

### Ethics Approval

The Institutional Review Board of the Institute of Tropical Medicine in Antwerp (867/13), the Ethics Committee of the University Teaching Hospital in Antwerp (13/14/147), the Lake Zone Institutional Review Board in Mwanza (MR/53/100/86) and the National Ethics Committee of the NIMR Coordinating Committee in Dar es Salaam (NIMR/HQ/R.8a/Vol.IX/1544) approved the study protocol. Permission to conduct the study was obtained from the Mwanza City Education Department and from Nyamagana and Ilemela Districts Education Authorities.

## Results

### Study Population

A total of 401 girls were enrolled in the study, of whom 2 girls were outside the age range of 17–18 years and excluded from the analyses. Results for STI testing, Nugent score and qPCR testing were available for 386 girls (Table 1). Of these girls, 216 (56%) were aged 17 years, 58 (15%) reported intravaginal cleansing, 9 (2%) reported receptive oral sex, and 163 (42%) reported that they had ever had penile-vaginal sex. Of the girls who reported having penile-vaginal sex, 69 (42%) reported having always used a condom with their last partner, and 64 (39%) reported that the difference in ages between the girls and her first sexual partner was 3 years or more.

**Table 1:** Socio-demographic characteristics, reported sexual history and hygiene management and reproductive tract infections among adolescent schoolgirls in Mwanza city, Tanzania (*N* = 386).

**Total**	**386**	**100%**
Age (years)		
17	215	56%
18	171	44%
Born in		
Mwanza region	290	75%
Other region	96	25%
Lives with		
Mother (+/– father/other person)	245	63%
Father (+/– other person, but not mother)	24	6%
Does not live with mother or father	117	30%
Number of people in household		
1–5	134	35%
6–7	130	34%
8 or more	122	32%
SES indicator (possessions)		
Car	24	6%
TV, but no car	165	43%
Cell phone, no car or TV	183	47%
None of the above	14	4%
Nights outside home, last 3 months		
None	340	88%
One or more	46	12%
Menstrual hygiene management		
Reusable cloth	150	39%
Underpants	372	96%
Sanitary pads	282	73%
Tampons or toilet paper	3	1%
Intravaginal cleansing		
No cleansing	328	85%
Plain water	34	9%
Soap	22	6%
Cloth, cotton wool, detergents	2	1%
Method of cleaning after defecation		
Water only	342	89%
Toilet paper	31	8%
Other	13	3%
Direction of cleaning after defecation		
Front to back	294	76%
Back to front	92	24%
Ever touched a penis with her hands	22	6%
Man/boy ever touched her vagina with hands	35	9%
Ever had penis in her mouth	3	1%
Ever had receptive oral sex	9	2%
Ever has a penis rub against her genitals	9	2%
Ever had anal sex^a^	2	1%
Life-time sexual (penile-vaginal sex) partners		
None	223	58%
One	123	32%
Two	31	8%
Three or more	9	2%
Age of first sexual partner (years older than the girl at the time of sexual debut)^b^		
<1 year older	16	10%
1-2 years older	31	19%
2-3 years older	30	18%
>3 years older	64	39%
Don't know/no answer	22	13%
Condom use with current/latest partner^b^		
Never	67	41%
Some of the time	24	15%
Always	69	42%
Don't know/no answer	3	2%
Bacterial vaginosis (Nugent 7–10)	95	25%
Intermediate microbiota (Nugent 4–6)	29	7%
Vaginal yeast	21	5%
*Chlamydia trachomatis*	9	2%
*Neisseria gonorrhoeae*	8	2%
*Trichomonas vaginalis*	17	4%
*Mycoplasma genitalium*	9	2%
Active syphilis	0	0%
Human papillomavirus–any genotype	125	32%
Herpes simplex virus-2	9	2%
HIV^c^	3	1%

a *Missing data for one participant*.

b *Restricted to participants who reported having at least one sexual partner*.

c *Missing data for two participant*.

The overall prevalence of BV (Nugent score 7–10) was 25%: 33% among the girls who reported penile-vaginal sex; and 19% among the girls who reported no penile-vaginal sex. Intermediate microbiota (Nugent score 4–6) was found in 7.5% of the girls: 17% among the girls who reported penile-vaginal sex; and 3% among the girls who reported no penile-vaginal sex. Five percent of the girls had a candida infection. Overall, 2% of girls had gonorrhoea, 2% had chlamydia, 4% had trichomoniasis, and 2% had *M. genitalium* infection. None of the girls was diagnosed with active syphilis and 2% had IgG antibodies against HSV-2. HIV infection was detected in three girls (1%). The most common STI/RTI was any HPV (high or low risk) infection, with 125 (32%) girls infected. A total of 19 (9%) of the 223 participants who reported no penile-vaginal sex, tested positive for HSV-2, chlamydia, gonorrhoea, *M. genitalium* (*n* = 12) or Y-chromosome (*n* = 7).

### Prevalence of Bacterial Species

Table 2 presents the proportion of girls in which each of the bacterial species was found as well as the mean log_10_ concentrations of the species if present. In the 223 girls who reported that they had never had penile-vaginal sex, the most commonly found bacterial species were *L. iners* (80%), *L. crispatus* (75%) and *L. vaginalis* (74%), followed by *L. jensenii* (55%). Among the 163 girls who reported that they had penile-vaginal sex, the prevalence of *L. crispatus* (60%)*, L. vaginalis* (55%) and *L. jenseniii* (40%) was lower (*p* < 0.01). The BV associated bacteria *A. vaginae, G. vaginalis* and *P. bivia* were found in the girls who did not report penile-vaginal sex though in lower proportions than among the girls who reported sexual activity (*p* < 0.01). There was no association between the prevalence of *L. iners* (*p* = 0.20) and of *L. gasseri* (*p* = 0.64) and reported penile-vaginal sex. A similar pattern was seen after excluding the girls with STIs/RTIs (Supplementary Table 1).

**Table 2:** Presence of Lactobacillus spp and BV-associated microbiota among secondary school girls in Mwanza, Tanzania (*N* = 386).

		**All participants *N* = 386**	**No reported penile-vaginal sex *N* = 223**	**Reported penile-vaginal sex *N* = 163**	***p*-value**
*L. crispatus*	Present^a^ *N* %	266 (69)	168 (75)	98 (60)	0.001
	Mean conc^b^	8.3	8.6	7.8	<0.001
*L. iners*	Present *N* %	318 (82)	179 (80)	139 (85)	0.20
	Mean conc	7.7	7.6	8.0	<0.001
*L. jensenii*	Present *N* %	188 (49)	123 (55)	65 (40)	0.003
	Mean conc	6.6	6.7	6.5	0.26
*L. vaginalis*	Present *N* %	255 (66)	166 (74)	89 (55)	<0.001
	Mean conc	5.8	5.8	5.8	0.44
*L. gasseri*	Present *N* %	85 (22)	51 (23)	34 (21)	0.64
	Mean conc	5.9	5.9	5.9	0.94
*A. vaginae*	Present *N* %	168 (44)	76 (34)	92 (56)	<0.001
	Mean conc	6.9	6.8	6.9	0.63
*G. vaginalis*	Present *N* %	239 (62)	116 (52)	123 (75)	<0.001
	Mean conc	6.6	6.5	6.8	0.06
*P. bivia*	Present *N* %	197 (51)	98 (44)	99 (61)	0.001
	Mean conc	4.8	4.7	4.9	0.18

a *Proportion of samples with a concentration of bacteria. P-values were obtained with Chi square for the association presence of each bacteria and reported penile-vaginal sex*.

b *Mean log_10_ concentration (geq/ml), if species is present. P-value was obtained by t-test for the association between mean log_10_ concentration and reported penile-vaginal sex*.

**Figure 1:**
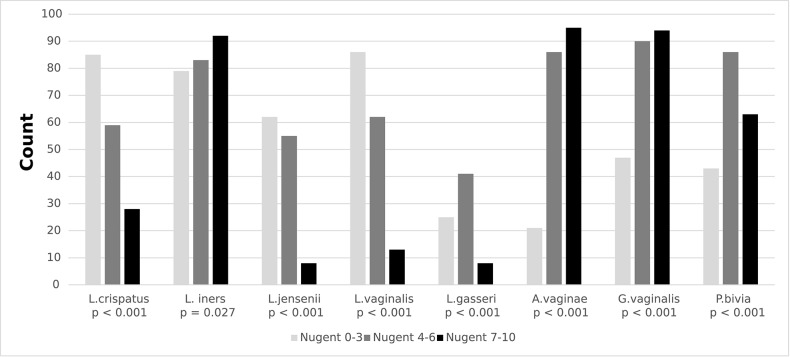
Presence of *Lactobacillus spp* and BV-associated microbiota by Nugent score among secondary school girls in Mwanza, Tanzania (*N* = 385).

The prevalence of *L. crispatus, L. jensenii* and of *L. vaginalis* decreased with increasing Nugent score (all *p* < 0.001), whereas the prevalence of *L. iners* increased with increasing Nugent score (*p* = 0.008) (Figure 1 and Supplementary Table 2). The prevalence of *L. gasseri* was low in girls with Nugent score 0–3 (25%) and even lower in girls with Nugent score 7–10 (8%, *p* = 0.004). The prevalence of *A. vaginae, G. vaginalis* and *P. bivia* increased with increasing Nugent score (all *p* < 0.001). Furthermore, the mean log_10_ concentrations of the *Lactobacillus spp*., if present, were lower in girls with BV compared with girls with Nugent score 0-3, except for *L. iners*. The mean log_10_ concentrations of *A. vaginae, G. vaginalis* and *P. bivia* were higher in girls with BV than in girls with Nugent score 0-3 (all *p* < 0.001).

The composition of BV-associated bacterial species for each category of Nugent score is shown in Supplementary Table 3. Samples with *G. vaginalis, A. vaginae* and *P. bivia* only (single pathogens) did not substantially contribute to the total Nugent-BV cases (0-1% of BV cases). Neither did samples with both *A. vaginae* and *P. bivia* (2%) nor samples with both *G. vaginalis* and *P. bivia* (2%). Samples with both *G. vaginalis* and *A. vaginae* contributed to 34% of the Nugent-BV cases, and samples with all three BV associated bacteria contributed 58% of the Nugent-BV cases. None of the three BV-associated bacteria were detected in two samples with Nugent score 7-10, but in one of these samples *L. iners* was detected.

### Factors Associated With the Presence of BV-Associated Bacteria

For each of the three BV-associated bacteria there was strong evidence of an association with penile-vaginal sex (Supplementary Tables 4A, 5A, 6A). In addition, as SES increased, there was a decreased odds of the presence of *G. vaginalis* (Supplementary Table 4A). Among the girls who reported that they had penile-vaginal sex an association was found between presence of *A. vaginae* and older age of the first sexual partner (Supplementary Table 5B); and between presence of *P. bivia* and life-time number of sexual partners (Supplementary Table 6B).

### Results of Fluorescence *in situ* Hybridisation (FISH) for *Gardnerella vaginalis* and *Atopobium vaginae*

**Figure 2:**
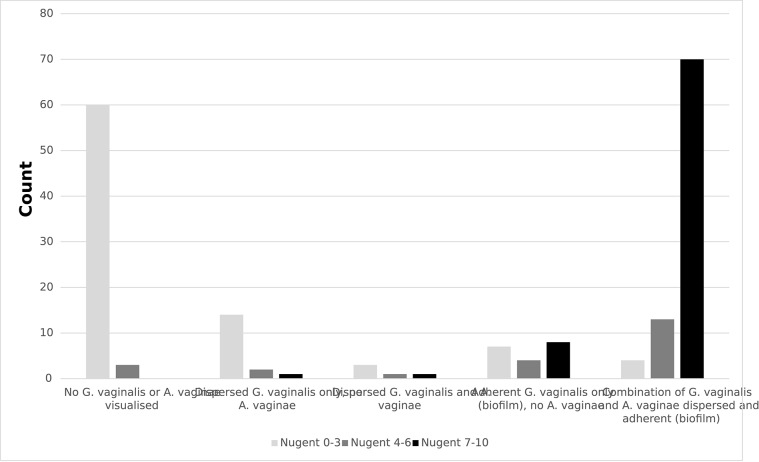
Results of fluorescence *in situ* hybridisation (FISH) for *Gardnerella vaginalis* and *Atopobium vaginae* by Nugent score among secondary school girls in Mwanza, Tanzania (*N* = 191; *p* < 0.001).

Of the 239 samples in which *G. vaginalis* was present, 191 (80%) had FISH results. Of these, 63 (33%) had no *G. vaginalis* or *A. vaginae* visualised, 17 (9%) had dispersed *G. vaginalis* only, 5 (3%) had dispersed *G. vaginalis* and *A. vaginae*, 19 (10%) had adherent *G. vaginalis* only (no adherent or dispersed *A. vaginae*), and 87 (46%) had a combination of dispersed or adherent *G. vaginalis* and *A. vaginae*. The latter category included 65 with both adherent *G. vaginalis* and *A. vaginae*, 20 with adherent *G. vaginalis* and dispersed *A. vaginae*, and 2 with dispersed *G. vaginalis* and adherent *A. vaginae*. There was 1 sample with *A. vaginae* dispersed only (no *G. vaginalis*); this sample was included in the category of dispersed *G. vaginalis* and *A. vaginae*. A Nugent score of 0–3 was more frequent among girls with no *G. vaginalis* or *A. vaginae* visualised compared to other categories. Conversely, a Nugent score of 7–10 was more frequent among girls with a combination of adherent and dispersed *G. vaginalis* and *A. vaginae* compared to other categories (χ^2^: *p* < 0.001; Figure 2). There was no difference in the FISH results by reported sexual activity (χ^2^: *p* < 0.211; Table 3).

**Table 3:** Results of fluorescence *in situ* hybridisation (FISH) for *Gardnerella vaginalis* and *Atopobium vaginae* by sexual activity among secondary school girls in Mwanza, Tanzania (*N* = 191).

	**Reported no penile-vaginal sex** ***N* = 90 (100%)**	**Reported penile-vaginal sex with at least one partner** ***N* = 101 (100%)**
No *G. vaginalis* or *A. vaginae* visualised	36 (40%)	27 (27%)
Dispersed *G. vaginalis* only, no *A. vaginae*	6 (7%)	11 (11%)
Dispersed *G. vaginalis* and *A. vaginae*	3 (3%)	2 (2%)
Adherent *G. vaginalis* only (biofilm), no *A. vaginae*	6 (7%)	13 (13%)
Combination of *G. vaginalis* and *A. vaginae* dispersed and adherent (biofilm)	39 (43%)	48 (47%)

*χ^2^(df) = 5.85(4), p = 0.211*.

### Results of the Detection of Sialidase A Gene Among Samples Positive for *G. vaginalis*

**Figure 3:**
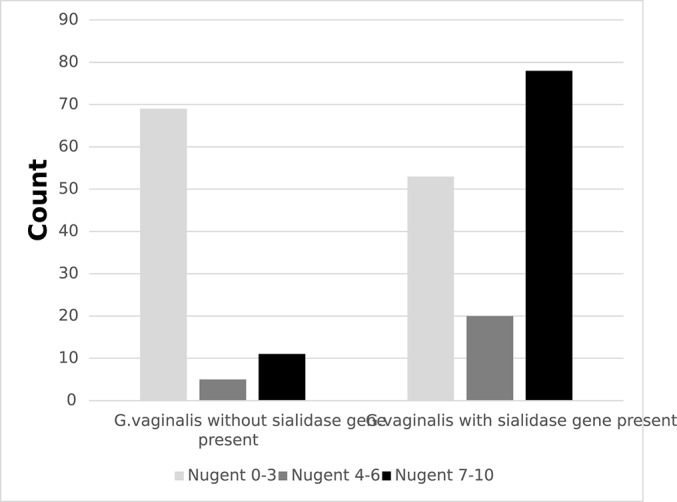
Frequencies of detection of *Gardnerella vaginalis* with or without putative sialidase A gene by Nugent score among secondary school girls in Mwanza, Tanzania (*N* = 236; *p* < 0.001).

Among the 239 girls with *G. vaginalis*, 236 had data for the presence of the sialidase A gene. Of these, 151 (63%) had *G. vaginalis* with the sialidase A gene. There was a positive correlation between the presence of sialidase A gene and high Nugent score; among the girls with *G. vaginalis*, Nugent score of 7–10 was more frequent among girls with sialidase A gene present compared to girls without sialidase A gene present (χ^2^: *p* < 0.001; Figure 3).

Girls who reported not having had penile-vaginal sex had a similar prevalence of sialidase A gene compared to girls reporting penile-vaginal sex with at least one partner (45 vs. 55%, respectively, *p* = 0.197, data not shown).

### Association Between Presence of the Sialidase A Gene, Biofilm and Non-optimal Microbiota

Of the 191 samples with *G. vaginalis* for which both FISH and sialidase A gene results were available, samples with adherent bacteria had higher proportions of sialidase positive *G. vaginalis* (χ^2^: *p* < 0.001; Table 4).

**Table 4:** Results of fluorescence *in situ* hybridisation (FISH) for *Gardnerella vaginalis* and *Atopobium vaginae* by presence of the sialidase A gene among 191 samples that have *G. vaginalis* present.

	***G.vaginalis*****; no sialidase gene** **(*****N*** **= 60)**		***G.vaginalis*****; sialidase gene present (*****N*** **= 131)**	
	**N Present (%)**	**Mean conc if present**	**N Present (%)**	**Mean conc if present**
No *G. vaginalis* or *A. vaginae* visualised	35 (58)	6.0	28 (21)	6.1
Dispersed *G. vaginalis* only, no *A. vaginae*	9 (15)	6.7	8 (6)	7.3
Dispersed *G. vaginalis* and *A. vaginae*	1 (2)	9.3	4 (3)	6.7
Adherent *G. vaginalis* only (biofilm), no *A. vaginae*	3 (5)	6.3	16 (12)	7.4
Combination of *G. vaginalis* and *A. vaginae* dispersed and adherent (biofilm)	12 (20)	7.2	75 (57)	7.6

*χ^2^(df) = 53.69(4), p < 0.001*.

Table 5 presents the association between presence of the sialidase A gene and non-optimal vaginal microbiota. The crude OR was 6.14 (95% CI 3.15–12.55), but after adjusting for FISH results, the OR was 2.29 (95% CI 0.77–6.65). The crude association between the FISH results and non-optimal vaginal microbiota was an OR of 83.12 (95% CI 33.74–232.60), and after adjusting for sialidase A gene, the OR was 67.00 (95% CI 26.72–190.53).

**Table 5:** Association between the presence of the sialidase A gene and results from fluorescence *in situ* hybridisation (FISH) with non-optimal vaginal microbiota (Nugent score 4–10) (*N* = 191).

	***N***	**Non-optimal microbiota Pos (%)**	**Odds ratio** **(95% CI)**	***p*-value**	**Adjusted odds ratio** **(95% CI)^a^**	***p*-value^b^**
Sialidase A gene						
Absent	60	15 (25%)	1	<0.001	1	0.134
Present	131	88 (67%)	6.14 (3.15–12.55)		2.29 (0.77–6.65)	
Fluorescence *in situ* hybridisation (FISH) for *Gardnerella vaginalis* and *Atopobium vaginae*						
Non-adherent	85	8 (9%)	1	<0.001	1	<0.001
Adherent (biofilm)	106	95 (90%)	83.12 (33.74–232.60)		67.00 (26.72–190.53)	

a *Variables were adjusted by the other variables in the model*.

b *P-values obtained with likelihood ratio tests*.

## Discussion

Few studies have examined the effect of sexual debut on the vaginal microbiota of young women. To our knowledge, this is the first paper to describe the vaginal microbiota, including vaginal biofilm among girls or young women in sub-Saharan Africa around the time of expected sexual debut. In this cross-sectional study, reported penile-vaginal sexual intercourse was associated with quantifiable changes in vaginal microbiota characterised by decreases in *Lactobacillus spp*. and increases in BV-associated bacteria. The prevalence of *L. crispatus* was higher than reported in previous studies among women in sub-Saharan Africa (Jespers et al., 2012). In our study population, samples with all three BV-associated bacteria present (*G. vaginalis, A vaginae* and *P. bivia*) made up the highest proportion of samples with Nugent-BV compared to samples with each bacterium alone or together in pairs, supporting the conceptual model presented by Muzny et al. (2019). In addition, non-*G. vaginalis* biofilm was rare, further supporting the hypothesis that colonisation with a virulent strain of *G. vaginalis* is a necessary first step in the pathogenesis in BV (Muzny et al., 2019). Among those with detectable *G. vaginalis*, two-thirds had sialidase A positive *G. vaginalis*, and presence of this gene was higher among women with adherent bacteria indicative of biofilm supporting *in vitro* evidence that sialidase A gene is an important virulence factor for *G. vaginalis* biofilm formation.

The high prevalence of *L. crispatus* among girls in our study is in contrast to other studies from sub-Saharan Africa and may reflect vaginal microbiota around the time of sexual debut. In a study investigating the vaginal microbiota among women from different risk groups in East and Southern Africa, the prevalence of *L. crispatus* was 38% among sexually active adolescents in Kenya compared to 60% in our study (Jespers et al., 2015). Several studies conducted in the United States and in Europe have compared the vaginal microbiota in women of different ethnic backgrounds, showing that a microbiota dominated by *L. crispatus* was less frequent in women of African ancestry than in Caucasian women, and that the microbiota of women of African ancestry was more often dominated by *L. iners* or bacterial species other than lactobacilli (Zhou et al., 2007; Ravel et al., 2011; Fettweis et al., 2014; Borgdorff et al., 2017). Yet, our study shows a similar prevalence of *L. crispatus* among girls who report not yet having penile-vaginal sex in Tanzania to sexually naïve Caucasian girls attending school in Belgium (69% in Tanzania and 68% in Belgium) (Jespers et al., 2016). These results are hypothesis generating, and longitudinal studies are needed to investigate if the *L. crispatus* prevalence diminishes with increased numbers of partners, uncircumcised partners or the initiation of intravaginal practices, which are more common in more sexually experienced women in the region (Gray et al., 2009; Allen et al., 2010).

Our data provide evidence for the role of penile-vaginal sex in the presence of *G. vaginalis, A. vaginae* and *P. bivia*: girls who reported having had at least one sexual partner had a higher prevalence and bacterial load, as well as strong independent associations between reported penile-vaginal sex and each bacterium. However, there was also evidence that some girls had prevalent *G. vaginalis, A. vaginalis* and *P. bivia* before reported sexual debut. This is consistent with longitudinal studies among adolescent girls in the US, Australia and in Belgium: *G. vaginalis* and *A. vaginae* were detected among some girls with no reported sexual experience, and the initiation of penile-vaginal sex was associated with increased presence of these BV-associated bacteria (Fethers et al., 2012; Mitchell et al., 2012; Jespers et al., 2016). An Australian study also found that engaging in unprotected penile-vaginal sex was associated with having multiple strains of *G. vaginalis*, including more virulent strains, such as *G. vaginalis* clade 4 which has been shown to develop a biofilm, produce the toxin vaginolysin, and express sialidase activity. In our study, we saw no difference in the FISH results or sialidase A positive *G. vaginalis* between girls reporting sexual activity vs. girls not. However, all these results must be considered in the context of underreporting of sexual activity. We found that 9% of girls who did not report penile-vaginal sex had a laboratory confirmed STI or tested positive on the Y-chromosome. Underreporting of sexual behaviour among adolescents has been well-documented internationally especially during face-to-face interviews (Langhaug et al., 2010), and fears of stigmatization, school expulsion and punishment may be even more relevant in the setting where this study was conducted (Houlihan et al., 2014).

Our FISH results are similar to other reports that show few or no samples with biofilm in the absence of *G. vaginalis* (Hardy et al., 2016). *In vitro* studies have shown that *G. vaginalis* is able to adhere to epithelial cells and displace *L. crispatus*, while other BV-associated bacteria, such as *A. vaginae* and *P. bivia* were outcompeted by protective lactobacilli (Rosca et al., 2019). Once a *G. vaginalis* biofilm is established, BV-associated bacteria can colonise and form synergistic interactions, by stimulating growth and upregulating key virulence factors such as genes encoding for sialidase. Indeed, our study shows a higher proportion of Nugent-BV among samples with both *G. vaginalis* and *A. vaginae* compared to biofilm with *G. vaginalis* alone, also seen in a previous study conducted in Rwanda (Hardy et al., 2016).

**Figure 4:**
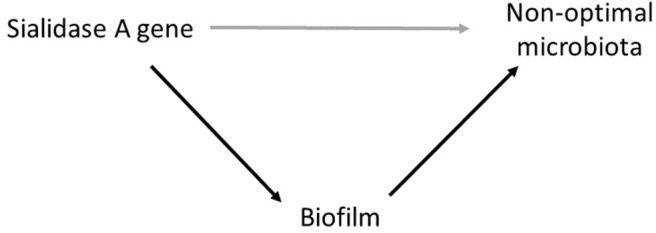
Conceptual model illustrating the relationship between Sialidase A gene, non-optimal microbiota (Nugent 4–10) and biofilm in which biofilm lies on the causal pathway between the Sialidase A gene and non-optimal microbiota.

There was a strong association between the samples with adherent bacteria indicative of biofilm and non-optimal microbiota. This was expected as “clue cells,” one of the criteria for BV diagnosis with Amsel's criteria, represent adherent bacteria. Importantly, this association was not substantially attenuated after adjustment for presence of the sialidase A gene. However, the association between sialidase A gene and non-optimal microbiota was substantially attenuated by adjusting for the FISH results, suggesting that biofilm lies on the causal pathway between sialidase A gene and non-optimal microbiota (Figure 4). In other words, much of the effect of sialidase production on non-optimal microbiota occurs through biofilm formation. This is supported by biological plausibility: sialidase destroys the protective mucus layer on the vaginal epithelium facilitating adhesion, successfully bypassing protective lactobacilli and providing a matrix in which secondary BV-associated bacteria can adhere and sequester. This is in alignment with earlier findings that showed an increased probability of having BV (diagnosed by Nugent scoring) when high concentration of *G. vaginalis* and the putative *G. vaginalis* sialidase A gene are present (Hardy et al., 2017b). The current study adds to the *in vivo* evidence of the importance of sialidase in a different population.

Our study has several limitations which call for caution in the interpretation of the results. Firstly, as noted above, there is evidence of underreporting of sexual activity. Furthermore, in the multivariable analyses of *G. vaginalis, A. vaginae and P. bivia*, we did not adjust each bacterium by the other two; therefore, it is possible that the associations with sexual activity is confounded by associations between the bacteria. To better answer the question of sexual acquisition of BV-associated bacteria, longitudinal studies are needed with validated behaviour measures and biomarkers for sexual activity. Secondly, we only tested a limited number of BV-associated bacteria by qPCR, and may have omitted a range of bacteria associated with enhanced vulnerability to HIV and other STIs (Eastment and Mcclelland, 2018). Our study did not test for sub-groups, clades or strains within *G. vaginosis* which may explain the difference in virulence (Vaneechoutte et al., 2019). Our study was also limited by FISH probes for *G. vaginalis* and *A. vaginae* only; future studies should investigate other species *in vivo* biofilm beyond *G. vaginalis* and *A. vaginae*, such as *P. bivia*, to better understand the interactions between vaginal microbiota. Importantly, while FISH is a useful method for identifying biofilm *in vivo* samples, the sensitivity is 66.7% (95% CI 54.5–77.1%) for *A. vaginae* 86.3% (95% CI: 77.4–92.2%) for *G. vaginalis* compared to qPCR (Hardy et al., 2015). To confirm the biofilm structure, future studies should complement the FISH analysis visualizing the bacteria constituting the biofilm with an assay detecting the extracellular matrix. Lastly, we tested samples for sialidase A gene, but did not measure sialidase production directly. While a previous study has shown that detection of sialidase A gene correlates very well with sialidase production (Lopes Dos Santos Santiago et al., 2011), another showed that some strains of *G. vaginalis* with sialidase A gene tested negative for sialidase production (Janulaitiene et al., 2018). We also did not investigate other virulence factors such as vaginolysin and others (Castro et al., 2019). A multi-omic approach would have provided a more comprehensive description of the interaction between the human host and microbiome, including identifying species/strains, genes, proteins, metabolites, and functional pathways.

In conclusion, this study among adolescent girls in Tanzania provides a snapshot of the vaginal microbiota around the time of expected sexual debut. These data challenge our understanding of *L. crispatus* prevalence among women with African ancestry, and confirms previous research showing a strong effect of sexual activity on the vaginal microbiota, although it remains unclear if this association is due to sexual transmission or sexual enhancement. This study also provides strong evidence for the role of *G. vaginalis* sialidase in biofilm formation and supports the conceptual model for the pathogenesis of BV that centres on the roles of virulent strains of *G. vaginalis*, as well as *P. bivia* and *A. vaginae*.

## Data Availability Statement

The datasets generated for this study are available on request to the corresponding author.

## Ethics Statement

The parents of all girls aged 17 and 18 years in forms 1-3 were informed about the study and asked for their written informed consent for their daughter to participate in the study if she was less than 18 years old. After parental consent, girls who were less than 18 years were asked for their written informed assent. Girls who were 18 years old were asked for their written informed consent.

## Author Contributions

SF, AB, TC, AA, VJ, JC, KB, RH, and DW-J contributed to the conception and design of the study. DW-J, JI, CH, and AA carried out the study. TS and CH performed the statistical analysis. AB and SF wrote the first draft of the manuscript. TC and LH wrote sections of the manuscript. All authors contributed to the manuscript revision, read, and approved the submitted version.

## Conflict of Interest

The authors declare that the research was conducted in the absence of any commercial or financial relationships that could be construed as a potential conflict of interest.
