# Supplementary material for: The Vaginal Microbiota Among Adolescent Girls in Tanzania Around the Time of Sexual Debut
Source: Front Cell Infect Microbiol. 2020 Jun 25;10:305. doi: 10.3389/fcimb.2020.00305 (PMC7330010; doi:10.3389/fcimb.2020.00305)
Supplement: Supplementary file 1 [file Data_Sheet_1.docx]

***Supplementary Material***

**Supplementary Table 1. Proportion of girls in which each of the species is present after excluding the girls with genital tract infection (N=225)**

|  |  | | **All participants N=225** | **No reported penile-vaginal sex  N=163** | **Reported penile-vaginal sex N=62** | **p-values^2^** |
| --- | --- | --- | --- | --- | --- | --- |
| *L. crispatus* | Present^1^ | N % | 176 (78) | 131 (80) | 45 (73) | 0.001 |
|  | Mean Conc^2^ | | 8.4 | 8.6 | 7.9 | < 0.001 |
| *L. iners* | Present^1^ | N % | 178 (79) | 128 (78) | 50 (81) | 0.202 |
|  | Mean Conc^2^ | | 7.6 | 7.5 | 7.9 | 0.001 |
| *L. jensenii* | Present^1^ | N % | 130 (58) | 101 (62) | 29 (47) | 0.003 |
|  | Mean Conc^2^ | | 6.6 | 6.6 | 6.7 | 0.256 |
| *L. vaginalis* | Present^1^ | N % | 168 (74) | 129 (79) | 39 (63) | < 0.001 |
|  | Mean Conc^2^ | | 5.8 | 5.9 | 5.7 | 0.438 |
| *L. gasseri* | Present^1^ | N % | 49 (22) | 35 (21) | 14 (23) | 0.638 |
|  | Mean Conc^2^ | | 5.8 | 5.8 | 5.9 | 0.939 |
| *A. vaginae* | Present^1^ | N % | 78 (35) | 52 (32) | 26 (42) | < 0.001 |
|  | Mean Conc^2^ | | 6.8 | 6.8 | 6.8 | 0.625 |
| *G. vaginalis* | Present^1^ | N % | 124 (55) | 86 (52) | 38 (61) | < 0.001 |
|  | Mean Conc^2^ | | 6.4 | 6.3 | 6.5 | 0.061 |
| *P. bivia* | Present^1^ | N % | 103 (46) | 68 (41) | 35 (56) | 0.001 |
|  | Mean Conc^2^ | | 4.6 | 4.7 | 4.5 | 0.184 |

^1^ Proportion of samples with a concentration of bacteria. P-values were obtained with Chi square for the association presence of each bacteria and reported penile-vaginal sex. ^2^Mean of log_10_ concentration (geq/ml), if species is present. P-value was obtained by t-test for the association between mean log_10_concentration and reported penile-vaginal sex.

**Supplementary Table 2: Proportion of girls in which each of the species is present and concentration of the species by Nugent score (N=385)**

|  |  | | **Nugent 0-3 N=262** | **Nugent 4-6 N=28** | **Nugent 7-10 N=95** | **Test for trend p-values** |
| --- | --- | --- | --- | --- | --- | --- |
| *L. cripatus* | Present^1^ | N % | 222 (85) | 16 (59) | 27 (28) | < 0.001 |
|  | Mean Conc^2^ | | 8.8 | 7.4 | 5.2 | < 0.001 |
| *L. iners* | Present^1^ | N % | 209 (79) | 24 (83) | 87 (92) | 0.008 |
|  | Mean Conc^2^ | | 7.6 | 8 | 8 | 0.011 |
| *L. jensenii* | Present^1^ | N % | 164 (62) | 16 (55) | 8 (8) | < 0.001 |
|  | Mean Conc^2^ | | 6.6 | 6.7 | 5.6 | 0.045 |
| *L. vaginalis* | Present^1^ | N % | 227 (86) | 18 (62) | 12 (13) | < 0.001 |
|  | Mean Conc^2^ | | 5.9 | 5.8 | 5 | < 0.001 |
| *L. gasseri* | Present^1^ | N % | 65 (25) | 12 (41) | 8 (8) | 0.004 |
|  | Mean Conc^2^ | | 5.9 | 6.6 | 5 | 0.038 |
| *A. vaginae* | Present^1^ | N % | 54 (21) | 25 (86) | 90 (95) | < 0.001 |
|  | Mean Conc^2^ | | 5.5 | 6.8 | 7.7 | < 0.001 |
| *G. vaginalis* | Present^1^ | N % | 124 (47) | 26 (90) | 89 (94) | < 0.001 |
|  | Mean Conc^2^ | | 5.8 | 7.4 | 7.6 | < 0.001 |
| *P. bivia* | Present^1^ | N % | 114 (43) | 25 (86) | 60 (63) | < 0.001 |
|  | Mean Conc^2^ | | 4.5 | 5.3 | 5.1 | < 0.001 |

Note: one observation removed due to missing Nugent score. ^1^ Proportion of samples with a concentration of bacteria. P-values were obtained with Chi square test for trend for the association presence of each bacteria and reported penile-vaginal sex. ^2^Mean of log_10_ concentration (geq/ml), if species is present. P-value was obtained by ANOVA for the association between mean log_10_ concentration and categories of microbiota.

**Supplemental Table 3. Frequencies (column percentages) giving the composition of BV-associated bacteria by qPCR for each category of Nugent-BV among secondary school girls in Mwanza, Tanzania (N=385^1^)**

| **Species (count)** | **N** | **Total** | **Optimal vaginal microbiota** | **Non-optimal vaginal microbiota** | |
| --- | --- | --- | --- | --- | --- |
|  |  |  | **Nugent 0-3** | **Nugent 4-6** | **Nugent 7-10** |
| Total | 385* | 100% | 262 (100) | 28 (100) | 95 (100) |
| None | 94 | 24% | 92 (35) | 0 (0) | 2 (2) |
| *A.vaginae* only | 3 | 1% | 2 (1) | 0 (0) | 1 (1) |
| *G.vaginalis* only | 40 | 10% | 38 (15) | 1 (4) | 0 (0) |
| *P.bivia* only | 41 | 11% | 39 (15) | 1 (4) | 1 (1) |
| *A.vaginae* + *P.bivia* | 9 | 2% | 5 (2) | 2 (7) | 2 (2) |
| G.vaginalis + P.bivia | 43 | 11% | 39 (15) | 2 (7) | 2 (2) |
| G.vaginalis + A.vaginae | 52 | 13% | 17 (6) | 3 (11) | 32 (34) |
| G.vaginalis + A.vaginae + P.bivia | 104 | 27% | 30 (11) | 19 (68) | 55 (58) |

Notes: ^1^One case is missing due to missing Nugent score.

**Supplemental Table 4a. Factors associated with the presence of *Gardnerella vaginalis* among secondary school girls in Mwanza, Tanzania (N=386)**

|  | **N** | **N Pos (%)** | **Odds ratio**  **(95% CI)** | **p-values^3^** | **Adjusted odds ratio**  **(95% CI)^4^** | **p-values^3^** |
| --- | --- | --- | --- | --- | --- | --- |
| **Total** | 386 | 239 (62) |  |  |  |  |
| **SOCIO-DEMOGRAPHC FACTORS** | | | | | | |
| **Age (years)** |  |  |  |  |  |  |
| 17 | 216 | 133 (62) | 1 | 0.876 | 1 | 0.807 |
| 18 | 170 | 106 (62) | 1.03 (0.68 – 1.56) |  | 1.05 (0.69 – 1.60) |  |
| **SES indicator (possessions)^1^** |  |  |  |  |  |  |
| Car in household | 24 | 10 (42) | 0.67 (0.49 – 0.92) | 0.012 | 0.67 (0.49 – 0.92) | 0.012 |
| TV, but no car in household | 165 | 97 (59) |  |  |  |  |
| Cell phone, no car or TV in household | 184 | 123 (67) |  |  |  |  |
| None of the above | 13 | 9 (69) |  |  |  |  |
| **Lives with** |  |  |  |  |  |  |
| Mother (+/-father/other person) | 246 | 155 (63) | 1 | 0.202 | 1 | 0.316 |
| Father (+/-other person, but not mother) | 24 | 18 (75) | 1.76 (0.67 – 4.60) |  | 1.88 (0.71 – 4.96) |  |
| Does not live with mother or father | 116 | 66 (57) | 0.77 (0.49 – 1.21) |  | 0.89 (0.56 – 1.42) |  |
| **BEHAVIOURAL FACTORS** | | |  |  |  |  |
| **Menstrual hygiene management^2^** |  |  |  |  |  |  |
| Sanitary pads or towels | 203 | 127 (63) | 1 | 0.912 | 1 | 0.876 |
| Cloth, toilet paper or pants | 179 | 111 (62) | 0.98 (0.65 – 1.48) |  | 0.95 (0.61 – 1.47) |  |
| **Intravaginal cleansing** |  |  |  |  |  |  |
| No cleansing | 328 | 196 (60) |  | 0.103 |  | 0.807 |
| Using water only | 34 | 25 (74) | 1.87 (0.85 – 4.14) |  | 1.18 (0.50 – 2.83) |  |
| Using other substances | 24 | 18 (75) | 2.02 (0.78 – 5.22) |  | 1.33 (0.48 – 3.68) |  |
| **Direction of cleaning after defecation** |  |  |  |  |  |  |
| Front to back | 294 | 181 (62) | 1 | 0.799 | 1 | 0.976 |
| Back to front | 92 | 58 (63) | 1.06 (0.66 – 1.73) |  | 1.01 (0.60 – 1.65) |  |
| **Man/boy touched vagina with hands** |  |  |  |  |  |  |
| No | 351 | 208 (59) | 1 | <0.001 | 1 | 0.151 |
| Yes | 35 | 31 (89) | 5.33 (1.84 – 15.42) |  | 2.20 (0.69 – 6.95) |  |
| **Receptive oral sex** |  |  |  |  |  |  |
| No | 377 | 230 (61) | 1 | 0.003 | 1 | 0.058 |
| Yes | 9 | 9 (100) | -- |  | -- |  |
| **Life-time sexual partners** |  |  |  |  |  |  |
| None | 223 | 116 (52) | 1 | <0.001 | 1 | 0.001 |
| One | 123 | 90 (73) | 2.52 (1.56 – 4.06) |  | 2.38 (1.45 – 3.91) |  |
| Two or more | 40 | 33 (83) | 4.35 (1.85 – 10.24) |  | 2.92 (1.16 – 7.34) |  |

SES=Socio-economic status. ^1^ SES indicator was fitted as a continuous covariate; the odds ratio of 0.67 estimates the decrease in odds of presence of *G vaginalis for* a one-step increase in SES score. ^2^ Missing data for some participants. ^3^P-values obtained with likelihood ratio tests. ^4^ All factors were adjusted for SES; behavioural factors were also adjusted for touching, receptive oral sex and life-time sexual partners.

**Supplemental Table 4b. Factors associated with the presence of *Gardnerella vaginalis* among sexually active secondary school girls in Mwanza, Tanzania (N=163)**

|  | **N** | **N Pos (%)** | **Odds ratio**  **(95% CI)** | **p-values^3^** | **Adjusted odds ratio**  **(95% CI)^4^** | **p-values^3^** |
| --- | --- | --- | --- | --- | --- | --- |
| **Total** | 163 | 123 (75) |  |  |  |  |
| **SOCIO-DEMOGRAPHC FACTORS** | | | | | | |
| **Age (years)** |  |  |  |  |  |  |
| 17 | 80 | 57 (71) | 1 | 0.22 |  |  |
| 18 | 83 | 66 (80) | 1.57 (0.76 – 3.22) |  |  |  |
| **SES indicator (possessions)^1^** |  |  |  |  |  |  |
| Car in household | 6 | 6 (100) | 0.68 (0.39 – 1.18) | 0.169 |  |  |
| TV, but no car in household | 73 | 57 (78) |  |  |  |  |
| Cell phone, no car or TV in household | 76 | 54 (71) |  |  |  |  |
| None of the above | 8 | 6 (75) |  |  |  |  |
| **Lives with** |  |  |  |  |  |  |
| Mother (+/-father/other person) | 98 | 76 (78) | 1 | 0.143 |  |  |
| Father (+/-other person, but not mother) | 12 | 11 (92) | 3.18 (0.39 – 26.02) |  |  |  |
| Does not live with mother or father | 53 | 36 (68) | 0.61 (0.29 – 1.29) |  |  |  |
| **BEHAVIOURAL FACTORS** | | |  |  |  |  |
| **Menstrual hygiene management^2^** |  |  |  |  |  |  |
| Sanitary pads or towels | 93 | 72 (77) | 1 | 0.504 | 1 | 0.555 |
| Cloth, toilet paper or pants | 70 | 51 (73) | 0.78 (0.38 – 1.60) |  | 0.80 (0.38 – 1.67) |  |
| **Intravaginal cleansing** |  |  |  |  |  |  |
| No cleansing | 121 | 86 (71) | 1 | 0.050 | 1 | 0.149 |
| Using water only | 23 | 21 (91) | 4.27 (0.95 – 19.2) |  | 3.3 (0.71 – 15.27) |  |
| Using other substances | 19 | 16 (84) | 2.17 (0.60 – 7.92) |  | 2.01 (0.54 – 7.45) |  |
| **Direction of cleaning after defecation** |  |  |  |  |  |  |
| Front to back | 118 | 90 (76) | 1 | 0.698 | 1 | 0.76 |
| Back to front | 45 | 33 (73) | 0.86 (0.39 – 1.88) |  | 0.88 (0.39 - 1.98) |  |
| **Man/boy touched vagina with hands** |  |  |  |  |  |  |
| No | 129 | 93 (72) | 1 | 0.039 | 1 | 0.239 |
| Yes | 34 | 30 (88) | 2.90 (0.95 – 8.83) |  | 1.94 (0.61 – 6.15) |  |
| **Receptive oral sex** |  |  |  |  |  |  |
| No | 155 | 115 (74) | 1 | 0.031 | 1 | 0.13 |
| Yes | 8 | 8 (100) | -- |  | -- |  |
| **Life-time sexual partners** |  |  |  |  |  |  |
| One | 123 | 90 (73) | 1 | 0.222 | 1 | 0.668 |
| Two or more | 40 | 33 (83) | 1.73 (0.70 – 4.29) |  | 1.23 (0.47 – 3.22) |  |
| **Condom use with latest partner^2^** |  |  |  |  |  |  |
| Always | 69 | 53 (77) | 1 | 0.761 | 1 | 0.65 |
| Not always | 91 | 68 (75) | 0.89 (0.43 – 1.86) |  | 0.84 (0.40 – 1.78) |  |
| **Age of first sexual partner^2^** |  |  |  |  |  |  |
| <1 year older | 16 | 12 (75) | 1 | 0.539 | 1 | 0.60 |
| 1–2 years older | 31 | 20 (65) | 0.61 (0.16 – 2.34) |  | 0.57 (0.15 – 2.25) |  |
| 2–3 years older | 30 | 24 (80) | 1.33 (0.32 – 5.64) |  | 1.26 (0.30 – 5.42) |  |
| 3 or more years older | 64 | 49 (77) | 1.09 (0.31 – 3.88) |  | 1.06 (0.24 – 3.12) |  |

SES=Socio-economic status. ^1^ SES indicator was fitted as a continuous covariate; the odds ratio of 0.68 estimates the decrease in odds of presence of *G vaginalis for* a one-step increase in SES score. ^2^ Missing data for some participants. ^3^ P-values obtained with likelihood ratio tests. ^4^ Behavioural factors were adjusted for sexual touching with hands, receptive oral sex, and cleansing.

**Supplemental Table 5a**. **Factors associated with the presence of *Atopobium vaginae* among secondary school girls in Mwanza, Tanzania (N=386)**

|  | **N** | **N Pos (%)** | **Odds ratio**  **(95% CI)** | **P^3^** | **Adjusted odds ratio**  **(95% CI)^4^** | **P^3^** |
| --- | --- | --- | --- | --- | --- | --- |
| **Total** | 386 | 168 (44) |  |  |  |  |
| **SOCIO-DEMOGRAPHIC FACTORS** | | |  |  |  |  |
| **Age (years)** |  |  |  |  |  |  |
| 17 | 216 | 98 (45) | 1 | 0.409 |  |  |
| 18 | 170 | 70 (41) | 0.84 (0.56 – 1.27) |  |  |  |
| **SES indicator (possessions)^1^** |  |  |  |  |  |  |
| Car in household | 24 | 10 (42) | 0.95 (0.70 – 1.29) | 0.752 |  |  |
| TV, but no car in household | 165 | 71 (43) |  |  |  |  |
| Cell phone, no car or TV in household | 184 | 81 (44) |  |  |  |  |
| None of the above | 13 | 6 (46) |  |  |  |  |
| **Lives with** |  |  |  |  |  |  |
| Mother (+/-father/other person) | 246 | 104 (42) | 1 | 0.533 |  |  |
| Father (+/-other person, but not mother) | 24 | 13 (54) | 1.61 (0.70 – 3.74) |  |  |  |
| Does not live with mother or father | 116 | 51 (44) | 1.07 (0.69 – 1.67) |  |  |  |
| **BEHAVIOURAL FACTORS** | | |  |  |  |  |
| **Menstrual hygiene management^2^** |  |  |  |  |  |  |
| Sanitary pads or towels | 203 | 92 (45) | 1 | 0.501 | 1 | 0.565 |
| Cloth, toilet paper or pants | 179 | 75 (42) | 0.87 (0.58 – 1.31) |  | 0.88 (0,58 - 1,35) |  |
| **Intravaginal cleansing** |  |  |  |  |  |  |
| No cleansing | 328 | 137 (42) | 1 | 0.212 | 1 | 0.839 |
| Using water only | 34 | 17 (50) | 1.39 (0.69 – 2.83) |  | 0.85 (0.39 – 1.87) |  |
| Using other substances | 24 | 14 (58) | 1.95 (0.84 – 4.52) |  | 1.20 (0.48 – 2.97) |  |
| **Direction of cleaning after defecation** |  |  |  |  |  |  |
| Front to back | 294 | 132 (45) | 1 | 0.329 | 1 | 0.201 |
| Back to front | 92 | 36 (39) | 0.79 (0.49 – 1.27) |  | 0.72 (0.44 – 1.19) |  |
| **Man/boy touched vagina with hands** |  |  |  |  |  |  |
| No | 351 | 143 (41) | 1 | <0.001 | 1 | 0.214 |
| Yes | 35 | 25 (71) | 3.64 (1.69 – 7.80) |  | 1.73 (0.72 – 4.12) |  |
| **Receptive oral sex** |  |  |  |  |  |  |
| No | 377 | 161 (43) | 1 | 0.034 | 1 | 0.453 |
| Yes | 9 | 7 (78) | 4.7 (0.96 –  22.9) |  | 1.90 (0.34 – 10.75) |  |
| **Life-time sexual partners** |  |  |  |  |  |  |
| None | 223 | 76 (34) | 1 | <0.001 | 1 | 0.001 |
| One | 123 | 63 (51) | 2.03 (1.3 –  3.18) |  | 1.87 (1.18– 2.97) |  |
| Two or more | 40 | 29 (73) | 5.10 (2.42 – 10.77) |  | 3.90 (1.74 – 8.77) |  |

SES=Socio-economic status. ^1^ SES indicator was fitted as a continuous covariate; the odds ratio of 0.95 estimates the decrease in odds of presence of *A vaginae for* a one-step increase in SES score. ^2^ Missing data for some participants. ^3^ P-values obtained with likelihood ratio tests. ^4^Behavioural factors were adjusted for touching, receptive oral sex and life-time sexual partners.

**Supplemental Table 5b**. **Factors associated with the presence of *Atopobium vaginae* among sexually active secondary school girls in Mwanza, Tanzania (N=163)**

|  | **N** | **N Pos (%)** | **Odds ratio**  **(95% CI)** | **P^3^** | **Adjusted odds ratio**  **(95% CI)^4^** | **P^3^** |
| --- | --- | --- | --- | --- | --- | --- |
| **Total** | 163 | 92 (56) |  |  |  |  |
| **SOCIO-DEMOGRAPHIC FACTORS** | | |  |  |  |  |
| **Age (years)** |  |  |  |  |  |  |
| 17 | 80 | 45 (56) | 1 | 0.961 |  |  |
| 18 | 83 | 47 (57) | 1.02 (0.55 – 1.89 |  |  |  |
| **SES indicator (possessions)^1^** |  |  |  |  |  |  |
| Car in household | 6 | 5 (83) | 0.67 (0.41 –  1.1) | 0.11 |  |  |
| TV, but no car in household | 73 | 43 (59) |  |  |  |  |
| Cell phone, no car or TV in household | 76 | 41 (54) |  |  |  |  |
| None of the above | 8 | 3 (38) |  |  |  |  |
| **Lives with** |  |  |  |  |  |  |
| Mother (+/-father/other person) | 98 | 56 (57) | 1 | 0.293 |  |  |
| Father (+/-other person, but not mother) | 12 | 9 (75) | 2.25 (0.57 – 8.82) |  |  |  |
| Does not live with mother or father | 53 | 27 (51) | 0.78 (0.40 – 1.52) |  |  |  |
| **BEHAVIOURAL FACTORS** | | |  |  |  |  |
| **Menstrual hygiene management^2^** |  |  |  |  |  |  |
| Sanitary pads or towels | 93 | 51 (55) | 1 | 0.634 | 1 | 0.7 |
| Cloth, toilet paper or pants | 70 | 41 (59) | 1.16 (0.62 – 2.18) |  | 1.16 (0.55 – 2.42) |  |
| **Intravaginal cleansing** |  |  |  |  |  |  |
| No cleansing | 121 | 65 (54) | 1 | 0.429 | 1 | 0.394 |
| Using water only | 23 | 14 (61) | 1.34 (0.54 – 3.33) |  | 0.53 (0.17 – 1.65) |  |
| Using other substances | 19 | 13 (68) | 1.87 (0.67 – 5.23) |  | 1.55 (0.43 – 5.55) |  |
| **Direction of cleaning after defecation** |  |  |  |  |  |  |
| Front to back | 118 | 69 (58) | 1 | 0.398 | 1 | 0.967 |
| Back to front | 45 | 23 (51) | 0.74 (0.37 – 1.48) |  | 1.02 (0.44 – 2.37) |  |
| **Man/boy touched vagina with hands** |  |  |  |  |  |  |
| No | 129 | 68 (53) | 1 | 0.058 | 1 | 0.145 |
| Yes | 34 | 24 (71) | 2.15 (0.95 – 4.86) |  | 2.08 (0.77 – 5.64) |  |
| **Receptive oral sex** |  |  |  |  |  |  |
| No | 155 | 86 (55) | 1 | 0.264 | 1 | 0.869 |
| Yes | 8 | 6 (75) | 2.41 (0.47 – 12.3) |  | 1.17 (0.18 – 7.85) |  |
| **Life-time sexual partners** |  |  |  |  |  |  |
| One | 123 | 63 (51) | 1 | 0.016 | 1 | 0.107 |
| Two or more | 40 | 29 (73) | 2.51 (1.15 – 5.47) |  | 2.21 (0.82 – 5.92) |  |
| **Condom use with latest partner^2^** |  |  |  |  |  |  |
| Always | 69 | 34 (49) | 1 | 0.122 | 1 | 0.367 |
| Not always | 91 | 56 (62) | 1.65 (0.87 – 3.10) |  | 1.41 (0.67 - 2.95) |  |
| **Age of first sexual partner^2^** |  |  |  |  |  |  |
| <1 year older | 16 | 8 (50) | 1 | 0.002 | 1 | 0.002 |
| 1–2 years older | 31 | 9 (29) | 0.41 (0.12 – 1.43) |  | 0.36 (0.10 – 1.30) |  |
| 2–3 years older | 30 | 23 (77) | 3.29 (0.90 - 12.00) |  | 3.08 (0.83 – 11.46) |  |
| 3 or more years older | 64 | 38 (59) | 1.46 (0.49 – 4.39) |  | 1.11 (0.36 – 3.44) |  |

SES=Socio-economic status. ^1^ SES indicator was fitted as a continuous covariate; the odds ratio of 0.95 estimates the decrease in odds of presence of *A vaginae for* a one-step increase in SES score. ^2^ Missing data for some participants. ^3^P-values obtained with likelihood ratio tests. ^4^ Behavioural factors were adjusted for sexual touching with hands, lifetime sexual partners and age difference with first partner.

**Supplemental Table 6a**. **Factors associated with the presence of *Prevotella bivia* among secondary school girls in Mwanza, Tanzania (N=386)**

|  | **N** | **n Pos (%)** | **Odds ratio**  **(95% CI)** | **p-value^3^** | **Adjusted odds ratio**  **(95% CI)^4^** | **p-value^3^** |
| --- | --- | --- | --- | --- | --- | --- |
| **Total** | 386 | 197 (51) |  |  |  |  |
| **SOCIO-DEMOGRAPHIC FACTORS** | | |  |  |  |  |
| **Age (years)** |  |  |  |  |  |  |
| 17 | 216 | 107 (50) | 1 | 0.507 |  |  |
| 18 | 170 | 90 (53) | 1.15 (0.77 – 1.71) |  |  |  |
| **SES indicator (possessions)^1^** |  |  |  |  |  |  |
| Car in household | 24 | 13 (54) | 1.23 (0.91 – 1.67) | 0.171 |  |  |
| TV, but no car in household | 165 | 89 (54) |  |  |  |  |
| Cell phone, no car or TV in household | 184 | 91 (49) |  |  |  |  |
| None of the above | 13 | 4 (31) |  |  |  |  |
| **Lives with** |  |  |  |  |  |  |
| Mother (+/-father/other person) | 246 | 123 (50) | 1 | 0.500 |  |  |
| Father (+/-other person, but not mother) | 24 | 15 (63) | 1.67 (0.70 – 3.95) |  |  |  |
| Does not live with mother or father | 116 | 59 (51) | 1.04 (0.67 – 1.61) |  |  |  |
| **BEHAVIOURAL FACTORS** | | |  |  |  |  |
| **Menstrual hygiene management^2^** |  |  |  |  |  |  |
| Sanitary pads or towels | 203 | 100 (49) | 1 | 0.457 | 1 | 0.406 |
| Cloth, toilet paper or pants | 179 | 95 (53) | 1.16 (0.78 – 1.74) |  | 1.19 (0.79 – 1.80) |  |
| **Intravaginal cleansing** |  |  |  |  |  |  |
| No cleansing | 328 | 167 (51) | 1 | 0.97 | 1 | 0.463 |
| Using water only | 34 | 18 (53) | 1.08 (0.53 – 2.20) |  | 0.77 (0.36 – 1.66) |  |
| Using other substances | 24 | 12 (50) | 0.96 (0.42 – 2.21) |  | 0.60 (0.25 – 1.48) |  |
| **Direction of cleaning after defecation** |  |  |  |  |  |  |
| Front to back | 294 | 154 (52) | 1 | 0.345 | 1 | 0.248 |
| Back to front | 92 | 43 (47) | 0.80 (0.50 – 1.28) |  | 0.75 (0.46 – 1.22) |  |
| **Man/boy touched vagina with hands** |  |  |  |  |  |  |
| No | 351 | 173 (49) | 1 | 0.028 | 1 | 0.592 |
| Yes | 35 | 24 (69) | 2.24 (1.07 – 4.72) |  | 1.25 (0.55 – 2.88) |  |
| **Receptive oral sex** |  |  |  |  |  |  |
| No | 377 | 193 (51) | 1 | 0.689 | 1 | 0.103 |
| Yes | 9 | 4 (44) | 0.76 (0.20 – 2.88) |  | 0.28 (0.06 – 1.33) |  |
| **Life-time sexual partners** |  |  |  |  |  |  |
| None | 223 | 98 (44) | 1 | <0.001 | 1 | 0.004 |
| One | 123 | 69 (56) | 1.63 (1.05 – 2.54) |  | 1.58 (1.00 – 2.50) |  |
| Two or more | 40 | 30 (75) | 3.83 (1.78 – 8.21) |  | 3.49 (1.52 – 8.01) |  |

SES=Socio-economic status. ^1^ SES indicator was fitted as a continuous covariate; the odds ratio of 1.23 estimates the increase in odds of presence of *P bivia for* a one-step increase in SES score. ^2^ Missing data for some participants. ^3^ P-values obtained with likelihood ratio tests. ^4^ Behavioural variables were adjusted for sexual touching by hands and life-time number of sexual partners.

**Supplemental Table 6b**. **Factors associated with the presence of *Prevotella bivia* among sexually active secondary school girls in Mwanza, Tanzania (N=163)**

|  | **N** | **n Pos**  **(%)** | **Odds ratio**  **(95% CI)** | **p-value^3^** | **Adjusted odds ratio**  **(95% CI)^4^** | **p-value^3^** |
| --- | --- | --- | --- | --- | --- | --- |
| **Total** | 163 | 99 (61) |  |  |  |  |
| **SOCIO-DEMOGRAPHIC FACTORS** | | |  |  |  |  |
| **Age (years)** |  |  |  |  |  |  |
| 17 | 80 | 45 (56) | 1 | 0.249 |  |  |
| 18 | 83 | 54 (65) | 1.45 (0.77 – 2.72) |  |  |  |
| **SES indicator (possessions)^1^** |  |  |  |  |  |  |
| Car in household | 6 | 3 (50) | 1.05 (0.65 – 1.7) | 0.85 |  |  |
| TV, but no car in household | 73 | 45 (62) |  |  |  |  |
| Cell phone, no car or TV in household | 76 | 46 (61) |  |  |  |  |
| None of the above | 8 | 5 (63) |  |  |  |  |
| **Lives with** |  |  |  |  |  |  |
| Mother (+/-father/other person) | 98 | 60 (61) | 1 | 0.157 |  |  |
| Father (+/-other person, but not mother) | 12 | 10 (83) | 3.17 (0.66 – 15.25) |  |  |  |
| Does not live with mother or father | 53 | 29 (55) | 0.77 (0.39 – 1.51) |  |  |  |
| **BEHAVIOURAL FACTORS** | | |  |  |  |  |
| **Menstrual hygiene management^2^** |  |  |  |  |  |  |
| Sanitary pads or towels | 93 | 53 (57) | 1 | 0.258 | 1 | 0.36 |
| Cloth, toilet paper or pants | 70 | 46 (66) | 1.45 (0.76 – 2.75) |  | 2.27 (1.02 – 5.07) |  |
| **Intravaginal cleansing** |  |  |  |  |  |  |
| No cleansing | 121 | 74 (61) | 1 | 0.964 | 1 | 0.815 |
| Using water only | 23 | 14 (61) | 0.99 (0.40 – 2.46) |  | 0.91 (0.36 – 2.31) |  |
| Using other substances | 19 | 11 (58) | 0.87 (0.33 – 2.33) |  | 0.72 (0.26 – 1.99) |  |
| **Direction of cleaning after defecation** |  |  |  |  |  |  |
| Front to back | 118 | 76 (64) | 1 | 0.123 | 1 | 0.154 |
| Back to front | 45 | 23 (51) | 0.58 (0.29 – 1.16) |  | 0.6 (0.3 –  1.21) |  |
| **Man/boy touched vagina with hands** |  |  |  |  |  |  |
| No | 129 | 75 (58) | 1 | 0.18 | 1 | 0.46 |
| Yes | 34 | 24 (71) | 1.73 (0.76 – 3.91) |  | 1.38 (0.58 – 3.25) |  |
| **Receptive oral sex** |  |  |  |  |  |  |
| No | 155 | 95 (61) | 1 | 0.528 | 1 | 0.261 |
| Yes | 8 | 4 (50) | 0.63 (0.15 – 2.62) |  | 0.42 (0.09 –  1.9) |  |
| **Life-time sexual partners** |  |  |  |  |  |  |
| One | 123 | 69 (56) | 1 | 0.03 | 1 | 0.03 |
| Two or more | 40 | 30 (75) | 2.35 (1.06 – 5.22) |  | 2.35 (1.06 – 5.22) |  |
| **Condom use with latest partner^2^** |  |  |  |  |  |  |
| Always | 69 | 42 (61) | 1 | 0.956 | 1 | 0.694 |
| Not always | 91 | 55 (60) | 0.98 (0.52 – 1.86) |  | 0.88 (0.45 – 1.69) |  |
| **Age of first sexual partner^2^** |  |  |  |  |  |  |
| <1 year older | 16 | 9 (56) | 1 | 0.988 | 1 | 0.995 |
| 1–2 years older | 31 | 19 (61) | 1.23 (0.36 – 4.19) |  | 1.15 (0.33 – 3.94) |  |
| 2–3 years older | 30 | 18 (60) | 1.17 (0,34 – 3.99) |  | 1.03 (0.30 – 3.56) |  |
| 3 or more years older | 64 | 39 (61) | 1.21 (0.40 – 3.67) |  | 1.03 (0.33 – 3.18) |  |

SES=Socio-economic status. ^1^ SES indicator was fitted as a continuous covariate; the odds ratio of 1.05 estimates the increase in odds of presence of *P bivia for* a one-step increase in SES score. ^2^ Missing data for some participants. ^3^P-values obtained with likelihood ratio tests. ^4^ Behavioural variables were adjusted for life-time number of sexual partners.
